# Efficacy and safety of durvalumab rechallenge in advanced hepatocellular carcinoma patients refractory to prior anti-PD-1 therapy

**DOI:** 10.1007/s12072-024-10728-9

**Published:** 2024-11-23

**Authors:** Kuan-Chang Lai, Yen-Hao Chen, Yi‑Ping Hung, Nai-Jung Chiang, Ming‑Huang Chen, San-Chi Chen

**Affiliations:** 1 Department of Oncology, Taipei Veterans General Hospital, Taipei, Taiwan; 2 Division of Hematology-Oncology, Department of Internal Medicine, Kaohsiung Chang Gung Memorial Hospital and Chang Gung University College of Medicine, Kaohsiung, Taiwan; 3 School of Medicine, College of Medicine, Chang Gung University, Taoyuan, Taiwan; 4 School of Medicine, College of Medicine, National Yang Ming Chiao Tung University, Taipei, Taiwan; 5 Institute of Clinical Medicine, National Yang Ming Chiao Tung University, Taipei, Taiwan

**Keywords:** Hepatocellular carcinoma (HCC), Immune checkpoint inhibitor (ICI), Anti Programmed cell death protein 1 (anti-PD-1), Anti Programmed cell death ligand 1 (anti-PD-L1), Nivolumab, Durvalumab, Anti-PD-1 refractory, Rechallenge, Immune, Related adverse event (irAE), Predictor

## Abstract

**Background/purpose:**

Recently, anti-programmed cell death protein-1 (anti-PD-1) and anti-PD-L1 therapies were approved for hepatocellular carcinoma (HCC). However, the effectiveness of rechallenging with one immune checkpoint inhibitor (ICI) after failure of another remains unclear. This study explores the efficacy and safety of anti-PD-L1 rechallenge in patients who failed anti-PD-1 therapy.

**Methods:**

From January 2016 to December 2023, 65 advanced HCC patients previously treated with anti-PD-1 therapy were retrospectively enrolled and rechallenged with durvalumab (480 mg IV every 2 weeks).

**Results:**

Overall, 86.2% of patients received nivolumab and 13.8% pembrolizumab as prior anti-PD-1 therapy. The overall response rate (ORR) to durvalumab was 13.8%. Patients who responded to prior anti-PD-1 had a higher ORR compared to non-responders (31.3% vs. 8.7%, *p* = 0.04). Patients with any grade of immune-related adverse events (irAEs) from durvalumab had a higher ORR than those without irAEs (35.3% vs. 6.7%, *p* = 0.01). The median PFS was 5.4 months, and the median OS was 9.6 months. Responders to prior anti-PD-1 showed longer OS (33.9 vs. 8.2 months, *p* < 0.01) and a trend toward longer PFS (13.8 vs. 4.9 months, *p* = 0.07) compared to non-responders. Multivariate analysis identified prior anti-PD-1 response (HR: 0.31) as the only protective factor for death. Common irAEs were skin toxicity (13.8%) and hepatitis (7.7%); no correlation was found between irAEs from prior anti-PD-1 and durvalumab treatment.

**Conclusion:**

This study provides the first, concrete evidence that durvalumab rechallenge is effective for HCC patients who are refractory to anti-PD-1 therapy, especially for those who previously responded to anti-PD-1 treatment.

**Supplementary Information:**

The online version contains supplementary material available at 10.1007/s12072-024-10728-9.

## Introduction

Hepatocellular carcinoma (HCC), the most common primary liver cancer, ranks as the sixth most prevalent cancer worldwide [1]. It was responsible for over 900,000 new cases and more than 800,000 deaths in 2020 [2]. Historically, treatment options for HCC were limited to the targeted therapy sorafenib [3] and, despite the development of second-line agents such as regorafenib [4], cabozantinib [5], and ramucirumab [6], the prognosis for advanced HCC remains poor.

Recent advancements in immune checkpoint inhibitors (ICIs), including anti-cytotoxic T lymphocyte-associated antigen 4 (anti-CTLA-4) and anti-programmed cell death-(ligand)1 (anti-PD-(L)1), have become the standard treatment, improving outcomes for various cancers, including HCC. Notably, pembrolizumab and the combination of nivolumab with ipilimumab were approved in 2018 and 2020, respectively, for second-line treatment of HCC due to their breakthrough responses [7, 8]. Later, combination therapies such as atezolizumab (anti-PD-L1) with bevacizumab [9] and dual immunotherapy with durvalumab (anti-PD-L1) and tremelimumab (anti-CTLA-4) [10] were also validated for first-line treatment in HCC.

A clinical challenge emerges when patients do not respond to initial anti-PD-1 therapies, such as nivolumab or pembrolizumab; the effectiveness of subsequent treatments with newer combinations like atezolizumab/bevacizumab or durvalumab/tremelimumab, both anti-PD-L1-based therapies, is uncertain. Conversely, with anti-PD-L1 combinations now established as standard first-line therapy, the efficacy of subsequent anti-PD-1 therapy following failure of these initial treatments also remains uncertain. When first ICI therapy proves effective, it may suggest an inflamed tumor microenvironment conducive to successful rechallenging with another ICI. However, acquired resistance due to immune escape could potentially undermine the effectiveness of rechallenges. The answers to these queries are crucial yet currently unresolved.

Given the earlier approvals of nivolumab and pembrolizumab for HCC, we collected data from patients who had previously received anti-PD-1 therapy to explore the efficacy of subsequent anti-PD-L1 treatment. Rechallenging was limited to patients using durvalumab to specifically assess the effectiveness and side effects of anti-PD-L1 rechallenge. This design aims to elucidate the potential benefits and risks associated with anti-PD-L1 rechallenge in this patient population.

## Materials and methods

### Patient selection

This study was a retrospective collection of clinical, pathological, and image information retrieved from electronic medical records. It included a total of 65 HCC patients from Taipei Veterans General Hospital (Taipei, Taiwan), from January 2016 to December 2023. The criteria for patient enrollment included aged 18 years or older at the time of diagnosis; cases of advanced HCC, particularly among patients who had experienced anti-PD-1 immunotherapy, and subsequently received anti-PD-L1 with durvalumab treatment (480 mg intravenously, every 2 weeks). The diagnosis of HCC was established either through histopathological confirmation or clinical interpretation, following the criteria set by the American Association for the Study of Liver Diseases (AASLD) [11]. All the detailed information was available, including age, gender, etiologies, biochemistry and hematology data, underlying comorbidities, tumor marker, tumor stage, comprehensive treatment information, survival time following diagnosis, and cause of death were collected. The presence of underlying cirrhosis was confirmed either through histological examination or by clinical and/or imaging evidence, including ascites, varices, splenomegaly, and thrombosis in the hepatic or portal veins. This study was approved by the Institutional Review Board of Taipei Veterans General Hospital (TPEVGH IRB No.: 2024-06-018BC).

### Outcome assessment

Immune-related adverse events (irAEs) were assessed based on main organ involvement and grading by Common Terminology Criteria for Adverse Events (CTCAE) version 5.0. Two independent investigators evaluated the treatment outcomes, categorizing them into complete response (CR), partial response (PR), stable disease (SD), and progressive disease (PD), based on RECIST and modified RECIST (Response Evaluation Criteria in Solid Tumors, version 1.1) criteria. We classified overall response rate (ORR, proportion of patients with a best overall response of partial or complete response) using RECIST version 1.1 version criteria by specialist review.

### Statistical analysis

The patient’s baseline characteristics are presented by calculating the arithmetic mean, standard deviation, median, and interquartile range. The associations between durvalumab treatment responses across groups were analyzed using the Fisher exact test and Chi-square (*χ*^2^) test. The Kaplan–Meier method was used to analyze OS and PFS. Survival curves were evaluated for differences using the log-rank test. A Cox proportional-hazards model was utilized both to calculate hazard ratios with 95% confidence intervals and to evaluate the effects of prognostic factors on progression and death in univariate and multivariate analyses. We selected relevant factors or variables with *p* value less than 0.1 in the univariate analysis for the multivariate model. All the tests were two-sided, and a *p* value of less than 0.05 was considered to indicate statistical significance. All the statistical analyses were performed by IBM^®^ SPSS^®^, version 21.0 (IBM Corp. Released 2012. IBM SPSS Statistics for Windows, Version 21.0. Armonk, NY: IBM Corp.).

## Results

### Patient characteristics

A total of 65 patients who were diagnosed with HCC received durvalumab during the study period from January 2016 to December 2023. Among them, 48 patients were male. The mean age was 60.5 ± 11.6. Regarding the etiology of HCC, HBV-related HCC accounted for 43.1% (28/65), HCV-related HCC for 3.1% (2/65), and non-viral etiology for 53.8% (35/65). Approximately 76.7% of the patients were classified as Child–Pugh score (CPS) A, and 18.3% fell into the Child–Pugh score B category. Albumin–bilirubin (ALBI) grade 1 accounted for 39.1% of patients and ALBI grade 2 for 56.3%. Furthermore, a total of 27 patients (41.5%) were diagnosed to have cirrhosis, 28 patients (43.1%) with portal vein thrombosis and 69.2% already had extrahepatic metastasis. Regard to prior systemic treatment, 84.6% of patients have been experienced targeted therapy with lenvatinib, and 86.2% received anti-PD-1 therapy with nivolumab. Moreover, regarding the combination regimen with durvalumab, 63.1% of patients underwent combination treatment with lenvatinib and 16.9% of patients received regorafenib. The median duration between the administration of prior anti-PD-1 and durvalumab was 6 months (interquartile range: 3.0–14.5 months). The detailed demographic and clinical characteristics are shown in Supplementary Table 1. Regarding the CPS (with five missing data points) and the ALBI grade (with one missing data point), these missing data were excluded from the liver function analysis.

### Efficacy and associated factors

**Fig. 1:**
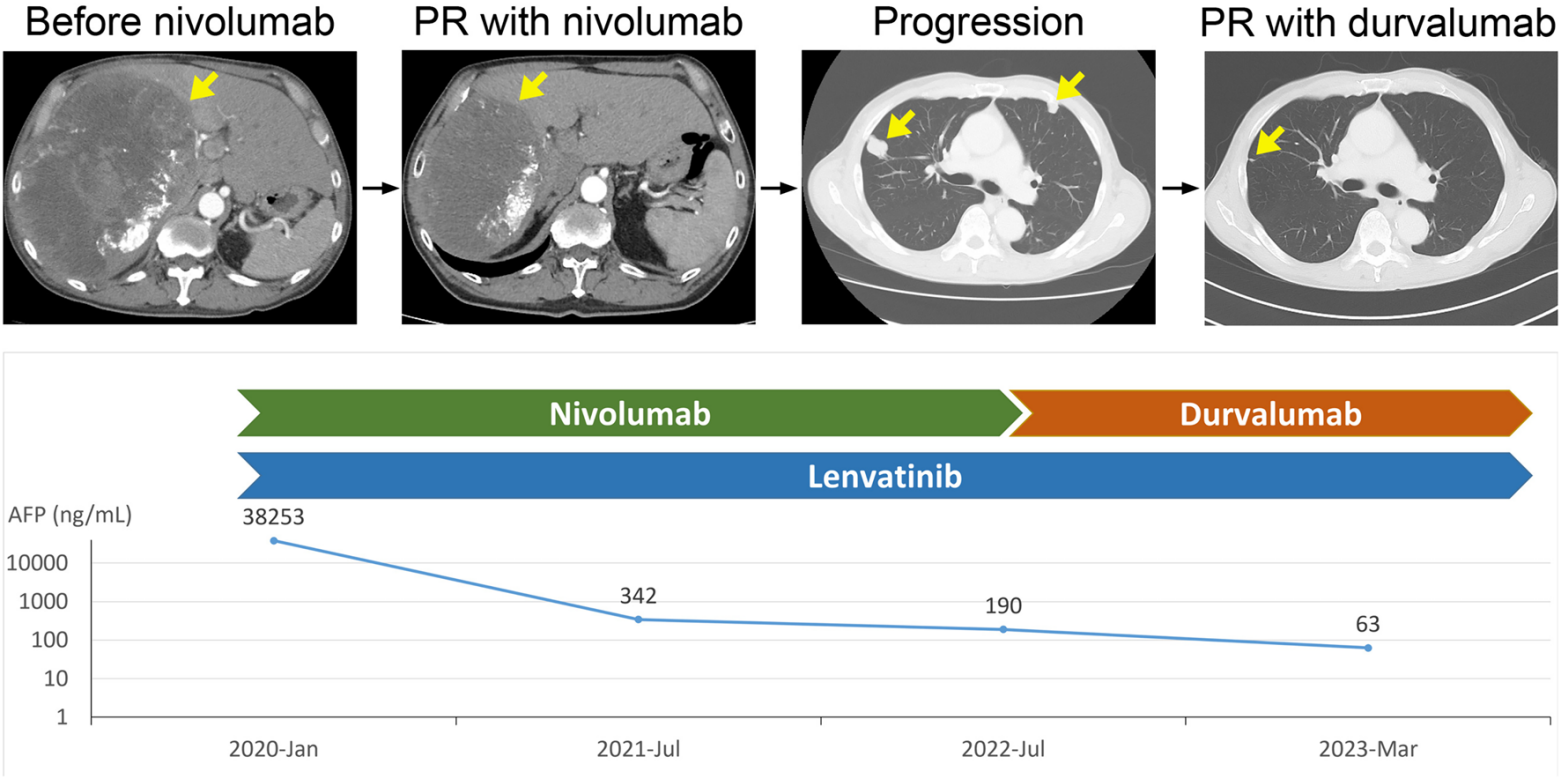
Time course of the presented case. The patient initially achieved a partial response with combined nivolumab and lenvatinib therapy, subsequently exhibited pulmonary metastasis progression, and then experienced tumor regression following the usage of durvalumab

The ORR to durvalumab was 13.8%, and disease control rate (DCR) was 70.8%, comprising a CR in 4.6%, a PR in 9.2%, and SD in 56.9%. Notably, two of the enrolled patients experienced clinical progression without image assessment, and one patient died from cancer within 2 months of initiating durvalumab treatment. These cases were classified as progressive disease. The waterfall plot categorized by etiologies is shown in Supplementary Fig. 1. Patients who responded to prior anti-PD-1 therapy had a higher ORR compared to non-responders (31.3% vs. 8.7%, *p* = 0.04). In addition, patients who experienced any grade of irAEs with durvalumab had a higher ORR compared to those without irAEs (35.3% vs. 6.7%, *p* = 0.01). However, factors such as sex, etiologies, liver function, tumor size, and intrahepatic tumor numbers were not associated with the response (Table 1). The ORR of different combination regimens with durvalumab is shown in Supplementary Table 2. We present a case in which the patient initially achieved a partial response with combined nivolumab and lenvatinib therapy and subsequently exhibited pulmonary metastasis progression with intrahepatic tumors remained stable. Then the pulmonary metastasis experienced tumor regression following the substitution of nivolumab with durvalumab (Fig. 1).

**Table 1:** Treatment response with durvalumab by RECIST 1.1 criteria

	CR	PR	SD	PD	ORR	*p* value
All population	4.6%	9.2%	56.9%	29.3%	13.8%	
Sex						
Male	4.4%	8.9%	57.8%	28.9%	13.3%	0.70
Female	5.9%	11.8%	64.7%	17.6%	17.7%	
Etiology						
HBV	2.3%	11.6%	62.8%	23.3%	14.0%	0.17
HCV	50.0%	0%	50.0%	0%	50.0%	
Non-viral	2.9%	5.7%	60.0%	31.4%	8.6%	
CPS classification						
A	4.4%	11.2%	62.2%	22.2%	15.6%	0.80
B	10.0%	10.0%	50.0%	30.0%	20.0%	
C	0%	0%	33.3%	66.7%	0%	
AIBI Score						
Grade I	4.3%	4.3%	78.3%	13.1%	8.7%	0.61
Grade II	5.6%	13.8%	50.0%	30.6%	19.4%	
Grade III	0%	0%	50.0%	50.0%	0%	
Intrahepatic tumor number						
1–5	7.1%	10.7%	64.3%	17.9%	17.9%	0.60
> 5–10	0%	0%	71.4%	28.6%	0%	
> 10	0%	11.8%	47.1%	41.1%	11.8%	
Infiltrating	0%	25.0%	50.0%	25.0%	25.0%	
Intrahepatic tumor size (cm)						
1–5	7.4%	11.1%	51.9%	29.6%	18.5%	0.62
> 5–10	0%	6.7%	73.3%	20.0%	6.7%	
> 10	0%	10.0%	60.0%	30.0%	10.0%	
Infiltrating	0%	25.0%	50.0%	25.0%	25.0%	
Metastasis						
Yes	2.2%	11.1%	60.0%	26.7%	13.3%	1.0
No	10.0%	5.0%	50.0%	35.0%	15.0%	
Prior anti-PD-1 agents						
Nivolumab	3.6%	8.9%	58.9%	28.6%	12.5%	0.33
Pembrolizumab	11.1%	11.1%	44.5%	33.3%	22.2%	
Prior anti-PD-1 response						
Responder	12.5%	18.8%	43.7%	25.0%	31.3%	0.04
Non-responder	2.2%	6.5%	65.2%	26.1%	8.7%	
irAE of durvalumab						
Nil	2.2%	4.4%	62.2%	31.2%	6.7%	0.01
Any grade	11.8%	23.5%	52.9%	11.8%	35.3%

### Progression-free survival and overall survival

**Fig. 2:**
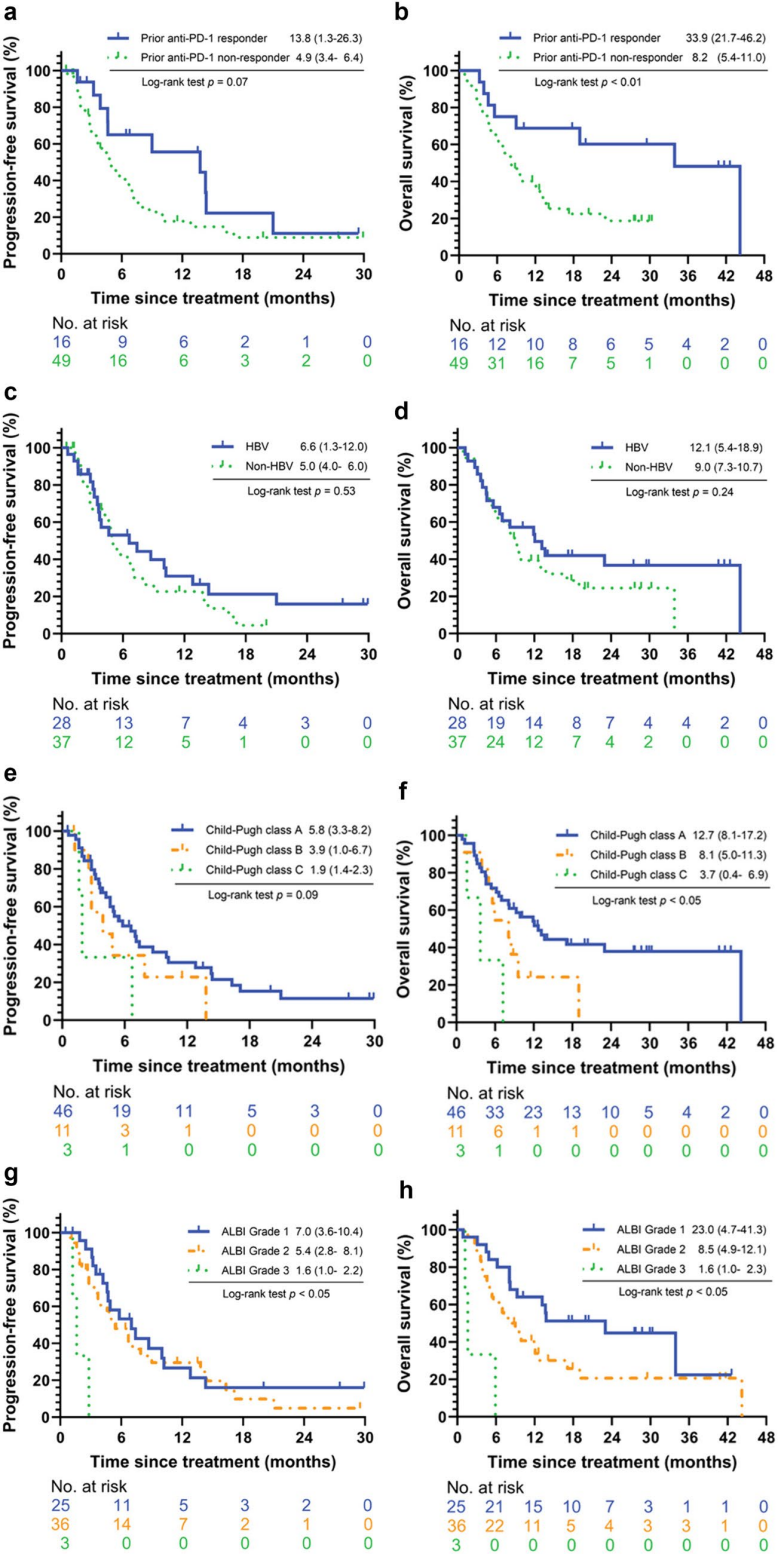
Progression-free survival and overall survival. The log-rank test indicated that patients who previously responded to anti-PD-1 therapy showed (**a**) a trend toward longer PFS (13.8 vs. 4.9 months, *p* = 0.07) and (**b**) significantly longer OS (33.9 vs. 8.2 months, p < 0.01) compared to non-responders. There was no significant difference in (**c**) PFS and (**d**) OS between patients with HBV and those without HBV. The difference in (**e**) PFS among different Child–Pugh classes was not significant, but was significant in (**f**) OS. On the other hand, different ALBI grades demonstrated significant differences in both (**g**) PFS and (**h**) OS

The median PFS of total population was 5.4 months [95% confidence interval (CI): 3.1–7.7], and the median OS 9.6 months [95% CI: 5.5–13.3]. The outcomes under durvalumab therapy and prior anti-PD-1 treatment were shown in swimmers’ plot (Supplementary Fig. 2). By prior anti-PD-1 response, the responders demonstrated longer OS (33.9 vs. 8.2 months, *p* < 0.01), and a trend of longer PFS (13.8 vs 4.9 months, *p* = 0.07) than those non-responders (Fig. 2A, B). According to the etiologies, patients with hepatitis B virus (HBV) infection showed no significant difference in PFS and OS when comparing with those in non-HBV patients (Fig. 2C, D). ALBI grades and CPS classes showed distinct prognostic impacts, with significant differences observed in survival outcomes. Patients across ALBI grades 1, 2, and 3 experienced varying PFS (7.0 vs. 5.4 vs. 1.6 months, *p* < 0.05) and OS (23.0 vs. 8.5 vs. 1.6 months, *p* < 0.05), highlighting ALBI grade as a critical prognostic indicator. While CPS class A patients demonstrated better OS compared to classes B and C (12.7 vs 8.1 vs 3.7 months, *p* < 0.05), the difference in PFS among CPS classes was not statistically significant (5.8 vs 3.9 vs 1.9 months, *p* = 0.09). The median PFS and OS of different combination regimens with durvalumab are shown in Supplementary Fig. 3.

### Prognostic factors for progression and death

Univariate analysis identified macrovascular invasion, intrahepatic tumor numbers > 10, alpha–fetoprotein (AFP) levels > 400 ng/mL, and CPS classes B and C as risk factors for progression, while a prior response to anti-PD-1 therapy was protective. However, in the multivariate analysis, none of these factors remained as independent predictors of risk (Table 2). Regarding risk factors for death, multivariate analysis indicated that age > 60 years [hazard ratio (HR): 2.55] and intrahepatic tumor numbers > 10 (HR: 4.06) were independent poor risk factors, while a prior response to anti-PD-1 therapy (HR: 0.31) was the only independent protective factor (Table 3).

**Table 2:** Risk factors for progression

Variables		Univariate		*p* value	Multivariate		*p* value
General factors		HR (95% CI)			HR (95% CI)		
Age	> 60 vs. ≤ 60	1.48	(0.82–2.66)	0.19			
Sex	Male vs. female	1.09	(0.58–2.03)	0.79			
HBsAg-positive	Yes vs. no	0.78	(0.42–1.44)	0.43			
Anti-HCV-positive	Yes vs. no	0.63	(0.15–2.62)	0.52			
Radiological factors							
Cirrhosis	Yes vs. no	1.17	(0.63–1.99)	0.71			
Ascites	Yes vs. no	1.25	(0.70–2.22)	0.45			
Macrovascular invasion	Yes vs. no	1.75	(0.98–3.11)	0.06	1.07	(0.45–2.51)	0.88
Metastasis	Yes vs. no	0.88	(0.48–1.61)	0.68			
Intrahepatic tumor numbers	> 10 vs. ≤ 10	1.90	(1.04–3.47)	0.04	1.42	(0.72–2.80)	0.32
Intrahepatic tumor size (cm)	> 10 vs. ≤ 10	1.45	(0.77–2.70)	0.25			
Previous local treatment							
Surgical tumor resection	Yes vs. no	0.66	(0.36–1.19)	0.16			
RFA	Yes vs. no	1.04	(0.50–2.14)	0.93			
TACE	Yes vs. no	0.75	(0.42–1.35)	0.34			
Radiotherapy	Yes vs. no	1.26	(0.53–2.98)	0.61			
Lab data							
ALT (U/L)	> 40 vs. ≤ 40	1.54	(0.88–2.71)	0.13			
ALBI grade	Grade 2, 3 vs. 1	1.49	(0.81–2.73)	0.20			
CPS class	Class B, C vs. A	1.83	(0.91–3.67)	0.09	1.62	(0.69–3.79)	0.27
AFP (ng/mL)	> 400 vs ≤ 400	2.27	(1.20–4.28)	0.01	1.83	(0.84–3.96)	0.13
Prior anti-PD-1 agents	Nivolumab vs. pembrolizumab	1.03	(0.44–2.42)	0.95			
Prior anti-PD-1 response	Responder vs. non-responder	0.53	(0.26–1.07)	0.08	0.65	(0.29–1.47)	0.30
Combined TKI of durvalumab	Lenvatinib vs. other TKIs	0.60	(0.33–1.11)	0.11			
	Regorafenib vs. other TKIs	0.57	(0.29–1.13)	0.11			

*ALT* alanine aminotransferase, ALBI grade albumin–bilirubin grade, *CPS class* Child–Pugh score class, *AFP* alpha-fetoprotein, *RFA* radiofrequency ablation, *TACE* transarterial chemoembolization, *TKI* tyrosine kinase inhibitor

**Table 3:** Risk factors for death

Variables		Univariate			Multivariate		
General factors		HR (95% CI)		*p* value	HR (95% CI)		*p* value
Age	> 60 vs. ≤ 60	2.18	(1.17–4.07)	0.01	2.55	(1.10–5.95)	0.03
Sex	Male vs. female	1.37	(0.68–2.77)	0.38			
HBsAg-positive	Yes vs. no	0.80	(0.42–1.55)	0.51			
Anti-HCV-positive	Yes vs. no	0.43	(0.06–3.17)	0.41			
Radiological factors							
Cirrhosis	Yes vs. no	1.32	(0.72–2.40)	0.37			
Ascites	Yes vs. no	1.56	(0.85–2.86)	0.15			
Macrovascular invasion	Yes vs. no	2.30	(1.24–4.25)	0.01	1.93	(0.76–4.93)	0.17
Metastasis	Yes vs. no	1.15	(0.59–2.24)	0.68			
Intrahepatic tumor numbers	> 10 vs. ≤ 10	3.56	(1.92–6.63)	< 0.01	4.06	(1.87–8.78)	< 0.01
Intrahepatic tumor size (cm)	> 10 vs. ≤ 10	1.39	(0.74–2.62)	0.31			
Previous local treatment							
Surgical tumor resection	Yes vs. no	0.65	(0.35–1.21)	0.17			
RFA	Yes vs. no	1.04	(0.50–2.17)	0.91			
TACE	Yes vs. no	0.74	(0.40–1.36)	0.33			
Radiotherapy	Yes vs. no	1.15	(0.51–2.57)	0.74			
Lab data							
ALT (U/L)	> 40 vs. ≤ 40	1.29	(0.70–2.37)	0.41			
ALBI grade	Grade 2, 3 vs. 1	2.18	(1.12–4.21)	0.02			
CPS class	Class B, C vs. A	2.36	(1.16–4.78)	0.02	*2.09	(0.89–4.88)	0.09
AFP (ng/mL)	> 400 vs ≤ 400	1.91	(0.98–3.71)	0.06	1.48	(0.66–3.31)	0.34
Prior anti-PD-1 agents	Nivolumab vs. pembrolizumab	0.74	(0.33–1.68)	0.48			
Prior anti-PD-1 response	Responder vs. non-responder	0.33	(0.14–0.79)	0.01	0.31	(0.10–0.99)	0.04
Combined TKI of durvalumab	Lenvatinib vs. other TKIs	0.61	(0.32–1.15)	0.13			
	Regorafenib vs. other TKIs	0.61	(0.29–1.29)	0.20			

*ALT* alanine aminotransferase, *ALBI grade* albumin–bilirubin grade, *CPS class* Child–Pugh score class, *AFP* alpha-fetoprotein, *RFA* radiofrequency ablation, *TACE* transarterial chemoembolization, *TKI* tyrosine kinase inhibitor

*Given the interaction between CPS class and ALBI grade, only CPS class was included in the multivariate analysis

### Immune-related adverse event

**Fig. 3:**
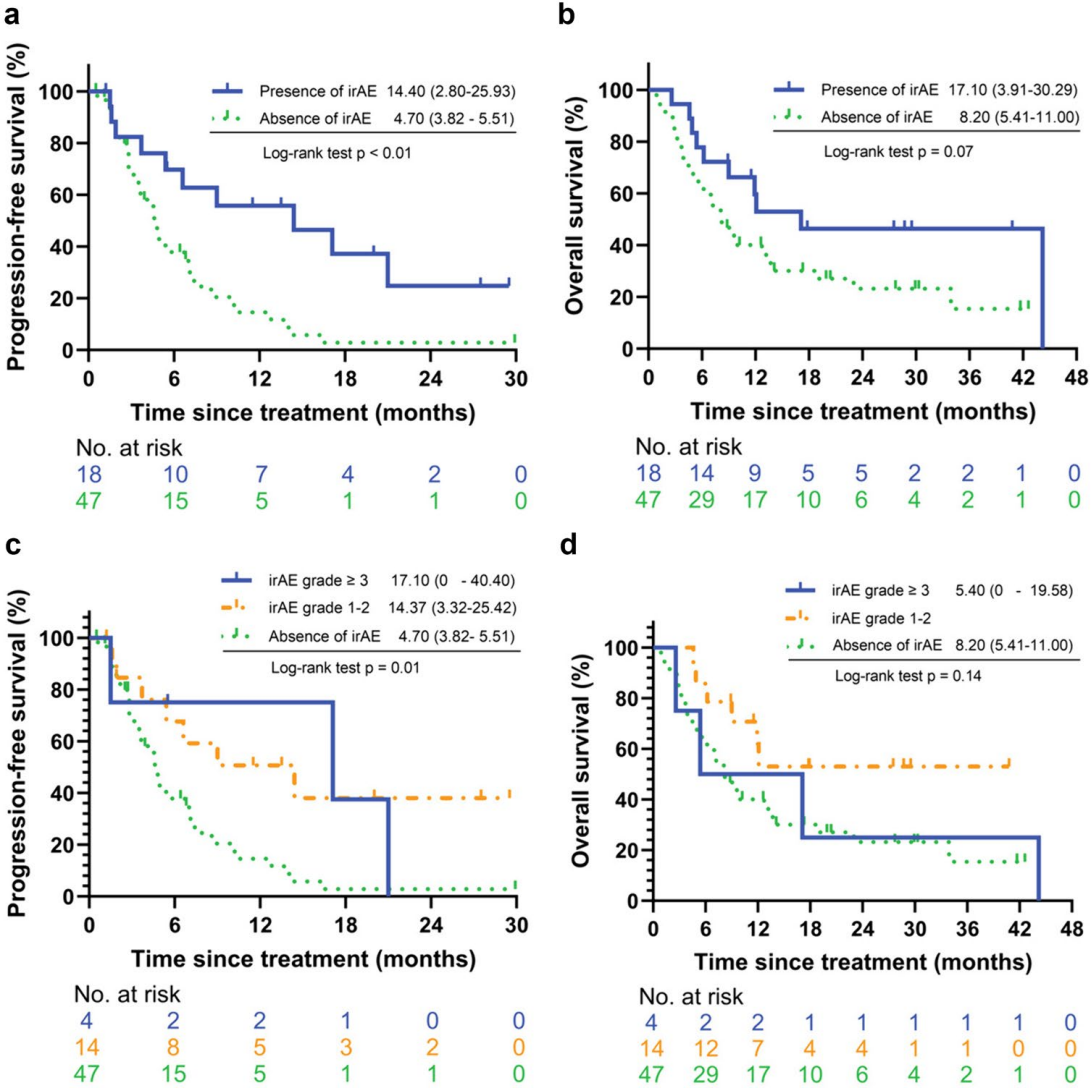
Progression-free survival and overall survival among different grading irAEs. The patients who experienced irAEs during durvalumab therapy had (**a**) significantly longer PFS (14.4 vs. 4.7 months, *p* < 0.01) and showed (**b**) a trend toward longer OS (17.1 vs. 8.2 months, *p* = 0.07) compared to those without irAEs. There was no significant difference in (**C**) PFS and (**D**) OS between low-grade and high-grade irAEs

In this cohort, the most common irAEs included skin toxicity (13.8%) and hepatitis (7.7%), with other irAEs such as hypothyroidism, fever, pancreatitis, pneumonitis, encephalopathy, diarrhea, diabetes, and myalgia occurring at an incidence rate of less than 5% (Supplementary Table 3). No correlation was observed between the occurrence of high-grade irAEs and prior anti-PD-1 treatment with subsequent durvalumab therapy (Supplementary Table 4). Similarly, no association was found for irAEs of any grade between prior anti-PD-1 and durvalumab treatment (Supplementary Table 5). The log-rank test revealed that patients who experienced irAEs during durvalumab therapy had significantly longer PFS (14.4 vs. 4.7 months, *p* < 0.01) and showed a trend toward longer OS (17.1 vs. 8.2 months, *p* = 0.07) compared to those without irAEs. No significant difference in PFS or OS was observed between patients with low-grade and high-grade irAEs (Fig. 3).

### Liver function monitoring

We utilized the ALBI grade to monitor changes in liver function following durvalumab treatment. Prior to initiating durvalumab, liver function assessments classified 39% of patients as ALBI grade 1, 56% as ALBI grade 2, and 5% as ALBI grade 3. After 4 weeks of durvalumab treatment, liver function assessment showed that 41% of patients were classified as ALBI grade 1, 54% as ALBI grade 2, and 5% as ALBI grade 3. After 8 weeks of treatment with durvalumab, 39% of patients were classified as ALBI grade 1, 53% as ALBI grade 2, and 8% as ALBI grade 3. The proportional changes in ALBI grade following durvalumab treatment are illustrated in Supplementary Fig. 4.

## Discussion

The major findings of this study include: (1) durvalumab is effective in anti-PD-1 refractory HCC; (2) a prior response to anti-PD-1 predicts the response and outcomes to durvalumab; (3) the irAEs from prior anti-PD-1 therapy showed no association with subsequent irAEs from durvalumab.

In HCC, the evidence supporting the strategy of rechallenging with a second ICI in patients who were previously refractory to an initial ICI is limited. Wong et al. reported a retrospective cohort study involving 25 HCC patients who had progressed on anti-PD-(L)1 therapy and were subsequently treated with a combination of ipilimumab and anti-PD-1 (nivolumab or pembrolizumab), demonstrating an ORR of 16%. This study found no significant difference in prognosis between cases of primary and acquired resistance to prior anti-PD-(L)1 treatment, marking it as the first to show that combining anti-CTLA-4 with anti-PD-1 could overcome resistance to prior anti-PD-(L)1 treatments [12]. Similarly, Alden et al. reported a retrospective study delivering salvage therapy with ipilimumab plus nivolumab to 32 patients who had progressed on prior anti-PD-(L)1 treatment, which showed an ORR of 22% [13]. Furthermore, Scheiner et al. described an international, retrospective multicenter study involving 58 HCC patients who received at least 2 lines of ICI-based therapies at 14 institutions. The therapies included ICI monotherapy, dual ICI regimens, and ICI combined with targeted therapies or anti-vascular endothelial growth factor (anti-VEGF) agents. The ORR for second ICI-based therapies reached up to 26%, despite the heterogeneity of the regimens used [14]. In summary, in HCC patients resistant to prior anti-PD-(L)1 treatments, rechallenge with dual immunotherapy comprising anti-PD-1 and anti-CTLA-4 has shown some evidence of antitumor efficacy. However, rechallenge with different anti-PD-(L)1 agents still lacks definitive data.

As for rechallenging with a second ICI in other cancer types, data remain sparse and the patient contexts are typically heterogeneous. Fujita et al. retrospectively analyzed the limited efficacy of salvage anti-PD-L1 with atezolizumab in 18 non-small lung cancer (NSCLC) patients previously treated with anti-PD-1 (nivolumab, pembrolizumab), noting that none achieved tumor shrinkage [15]. Similarly, Kitagawa et al. reported an ORR of 5.9% among 17 NSCLC patients undergoing interchange between anti-PD-1 (nivolumab and pembrolizumab) and anti-PD-L1 (atezolizumab) as rechallenge therapy, where 41.2% of cases showed PD-L1 expression greater than 1% [16]. Furthermore, a meta-analysis incorporating 18 studies of NSCLC involving patients previously treated with anti-PD-(L)1 with or without anti-CTLA-4, and subsequently rechallenged with anti-PD-(L)1 after disease progression, demonstrated a pooled ORR of 8%, indicating a modest benefit [17]. In metastatic renal cell carcinoma, another meta-analysis that included ten studies from prospective, retrospective, or ambispective designs revealed therapeutic benefits from reintroducing second-line ICI-containing treatments in patients previously treated with ICIs, with a pooled ORR of 19% [18]. In summary, evidence regarding the interchange between anti-PD-1 and anti-PD-L1 is limited and the available data appear controversial.

The mechanisms underlying primary and acquired resistance to ICIs are complex, and overcoming this resistance remains a significant challenge. Any disruption in the key steps of the immune response against cancer diminishes the effectiveness of ICIs. These key processes include the release of neoantigens from cancer cells, their capture and presentation by antigen-presenting cells, the recognition of these neoantigens and subsequent activation of CD8 + cytotoxic T cells, and the interaction of these T cells with malignant cells [19–21]. Among the critical elements of the immune mechanism, the upregulation of immune checkpoints such as CTLA-4, PD-(L)1, lymphocyte activation gene 3 (LAG-3), T cell immunoglobulin and mucin domain 3 (TIM-3), and V-domain Ig suppressor of T cell activation (VISTA) can inhibit interactions between T cells and malignant cells, contributing to the loss of T cell effector function [22]. Theoretically, when cancer becomes refractory to one type of ICI, other ICIs may still be effective. Clinical data reviewed earlier demonstrate that anti-CTLA-4 can effectively treat PD-(L)1 refractory cancers. In addition, relatlimab, a novel ICI targeting the LAG-3, was shown to have an ORR of 12.0% when combined with nivolumab in melanoma previously treated with anti-PD-(L)1, according to the phase I/IIa RELATIVITY-020 trial [23]. This trial offers a rationale for rechallenging with different ICI targets in resistant cases.

Anti-PD-1 and anti-PD-L1 inhibitors target the same signaling pathway and are approved for many of the same cancer types, often leading to their consideration as equivalent therapies. However, anti-PD-1 inhibitors block signals through both PD-L1 and PD-L2 ligands, adding a layer of complexity to their mechanism of action [24]. In contrast, Linhares et al. demonstrated that anti-PD-L1 inhibitors have lower half maximal effective concentrations (EC50) and are more potent than anti-PD-1 inhibitors, as evidenced by functional assays conducted in vitro [25]. Therefore, despite their similarities, significant differences between these drugs exist. These differences may partially explain why durvalumab, a PD-L1 inhibitor, shows some therapeutic effects in patients with HCC previously refractory to anti-PD-1 therapy in our study.

Regarding predictive markers, immunotherapy has shown particular effectiveness in treating HBV-related HCC. Subgroup analysis from the IMbrave150 study indicated that HBV-related HCC generally has better outcomes compared to the non-HBV group [26]. Similarly, the HIMALAYA study demonstrated that the STRIDE regimen resulted in more favorable outcomes for HBV-HCC [27]. However, our study did not find a significant difference in efficacy between HBV-related and non-HBV-related HCC, possibly due to the heavily pretreated patient population. As for liver function, several studies have identified the ALBI grade as a predictive marker in systemic treatments for HCC [28]. Our findings align with this, underscoring the importance of ALBI grade as a critical prognostic indicator in our cohort.

Currently, there are no standard therapies recommended by guidelines for HCC patients who have failed multiple lines of treatment. In our cohort, 76.9% of patients had previously received more than two lines of therapy, with all patients having been treated with anti-PD-1 therapy and at least one molecular targeted therapy. Although the combination of ICI and molecular targeted therapies lacks robust evidence from randomized controlled trials—evidenced by the failures of studies like LEAP-002 and COSMIC-312—meta-analyses have shown potential benefits from these combinations [29]. For example, a meta-analysis that included both randomized controlled trials and single-arm studies demonstrated that ICI combined with targeted therapy resulted in higher ORR and DCR, as well as longer PFS and OS, compared to ICI monotherapy. Similar findings were reported in meta-analyses focusing on lenvatinib combined with ICI and HCV-related HCC [30–32]. This evidence supports the rationale for using combination therapy in the real-world treatment of our heavily pretreated patients. In addition, the combination therapy of a TKI with an anti-PD1/PD-L1 agent is also being evaluated in the IMBRAVE-251 study. While the results are awaited, the trial design suggests that this regimen could be considered as a salvage treatment for patients previously treated with ICIs.

Our study is subject to several limitations. First, as a retrospective analysis, it may be affected by information bias and selection bias. However, in the absence of prospective clinical trials addressing this specific issue, retrospective analysis is the only feasible approach. Second, patient heterogeneity, particularly regarding the number of prior systemic treatments and the combinations with different targeted therapies, may introduce bias into the analysis of therapeutic efficacy. However, our analysis indicated that durvalumab combined with various targeted therapies consistently showed efficacy across the cohort. Moreover, the Cox regression model did not reveal any significant differences in efficacy between the different targeted therapies, suggesting that the treatment effects observed are consistent and not strongly influenced by the specific choice of targeted therapy. Nonetheless, these findings should be interpreted with caution, given the inherent challenges posed by patient heterogeneity. Finally, our study focused on anti-PD-L1 rechallenge for anti-PD-1 refractory HCC; whether the reverse sequence of administration could offer benefits remains unknown.

## Conclusion

This study provides proof to the concept that anti-PD-L1 rechallenge is efficacious for anti-PD-1 refractory HCC, particularly in patients who previously responded to anti-PD-1 therapy. Treatment with durvalumab resulted in improved response and prognosis, offering a rationale for considering anti-PD-L1 rechallenge in clinical practice. However, prospective clinical trials are necessary to validate these findings.

## Supplementary Information

Below is the link to the electronic supplementary material.

Supplementary file1 Supplementary Figure 1. Waterfall plot. The waterfall plot illustrates the greatest percentage change in the sum of the longest diameters of target lesions according to RECIST criteria. (Blue represents HBV, Red denotes HCV, and Green indicates non-viral cases.) (TIF 1008 KB)

Supplementary file2 Supplementary Figure 2. Swimmers’ plot of prior anti-PD-1 and durvalumab treatment. Swimmers’ plot depicts PFS of prior anti-PD-1 treatment and durvalumab by each case. The outcomes under durvalumab therapy are also shown. (TIF 3414 KB)

Supplementary file3 Supplementary Figure 3. Progression-free survival and overall survival among different combination regimens with durvalumab. There was no significant difference in (A) PFS and (B) OS among different combination regimens with durvalumab. (TIF 652 KB)

Supplementary file4 Supplementary Figure 4. Liver function monitoring. The proportional changes in ALBI grade following durvalumab treatment at baseline, and after 4 and 8 weeks. (TIF 242 KB)

Supplementary file5 (DOCX 31 KB)

## Data Availability

Data are available by requesting.
