# Supplementary figures and images for: Efficacy and safety of durvalumab rechallenge in advanced hepatocellular carcinoma patients refractory to prior anti-PD-1 therapy
Source: Hepatol Int. 2024 Nov 23;18(6):1804–14. doi: 10.1007/s12072-024-10728-9 (PMC11632046; doi:10.1007/s12072-024-10728-9)

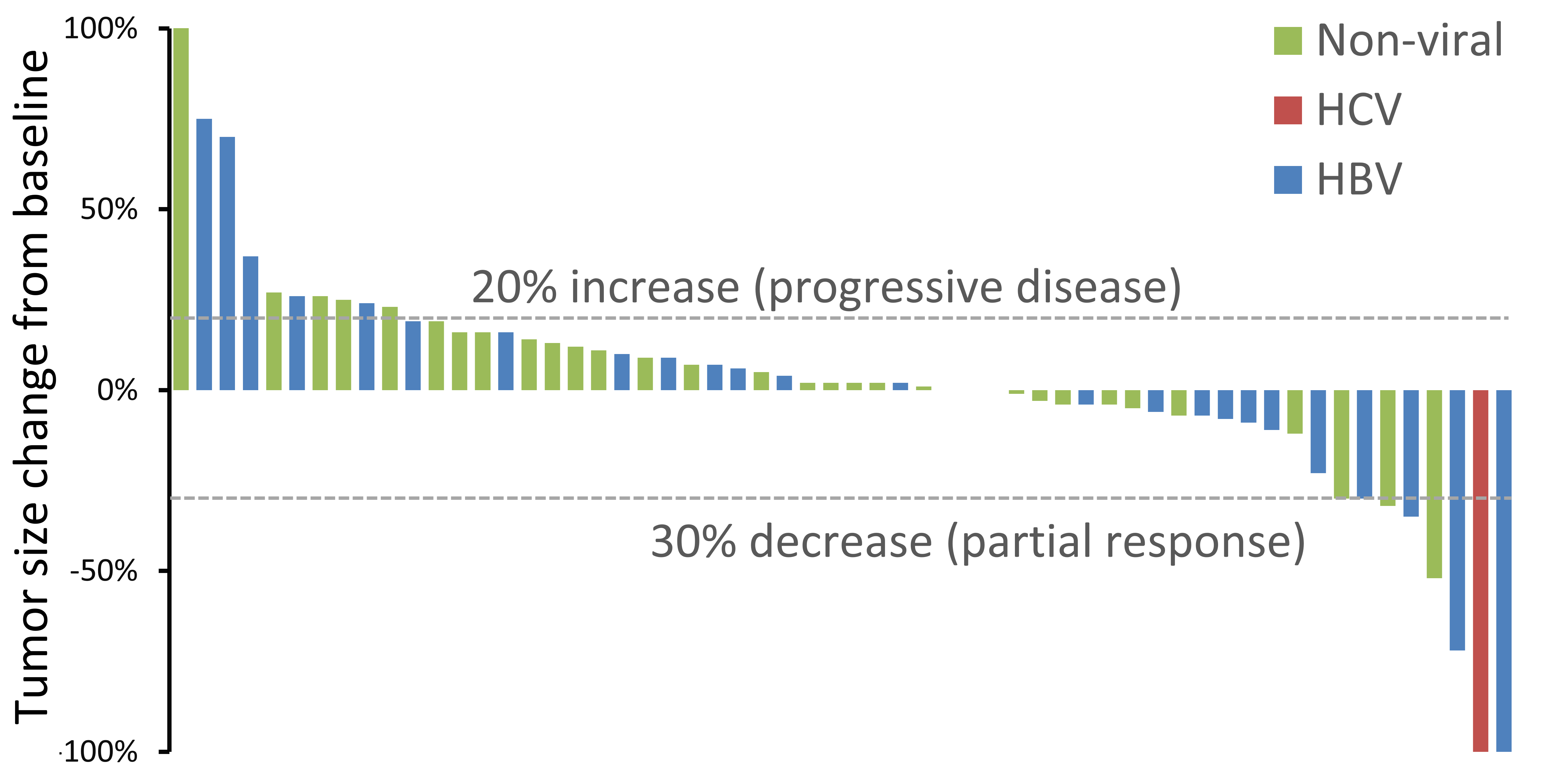

Supplement: Supplementary file 1 — Supplementary file1 Supplementary Figure 1. Waterfall plot. The waterfall plot illustrates the greatest percentage change in the sum of the longest diameters of target lesions according to RECIST criteria. (Blue represents HBV, Red denotes HCV, and Green indicates non-viral cases.) (TIF 1008 KB) [file 12072_2024_10728_MOESM1_ESM.tif]

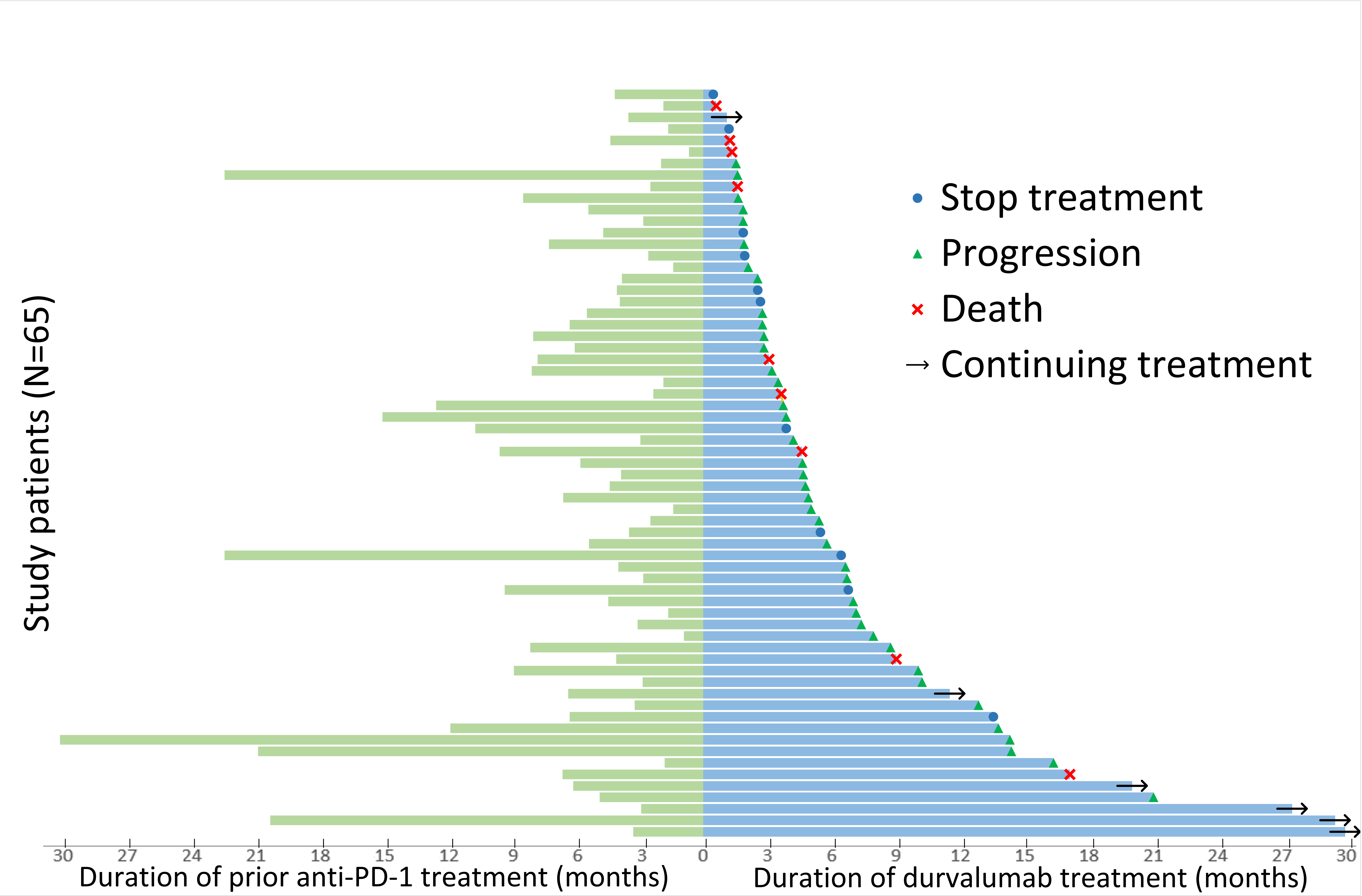

Supplement: Supplementary file 2 — Supplementary file2 Supplementary Figure 2. Swimmers’ plot of prior anti-PD-1 and durvalumab treatment. Swimmers’ plot depicts PFS of prior anti-PD-1 treatment and durvalumab by each case. The outcomes under durvalumab therapy are also shown. (TIF 3414 KB) [file 12072_2024_10728_MOESM2_ESM.tif]

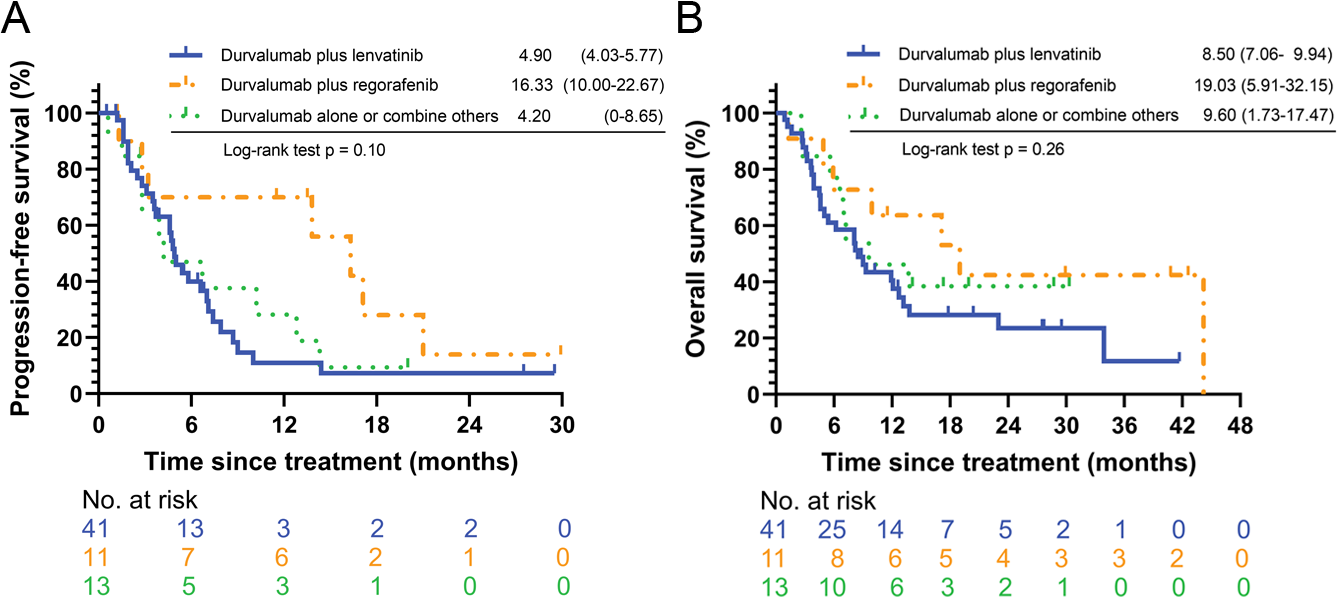

Supplement: Supplementary file 3 — Supplementary file3 Supplementary Figure 3. Progression-free survival and overall survival among different combination regimens with durvalumab. There was no significant difference in (A) PFS and (B) OS among different combination regimens with durvalumab. (TIF 652 KB) [file 12072_2024_10728_MOESM3_ESM.tif]

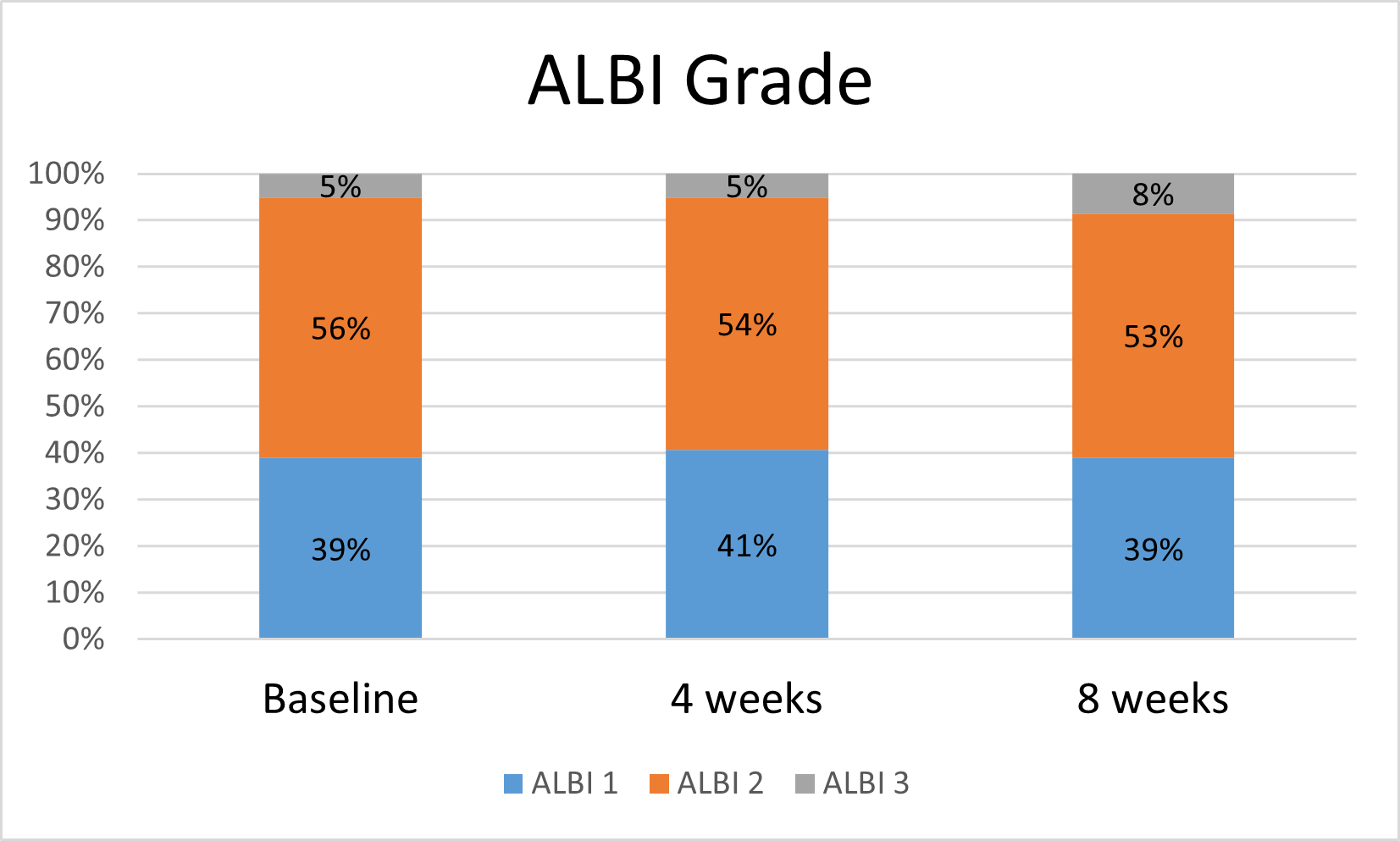

Supplement: Supplementary file 4 — Supplementary file4 Supplementary Figure 4. Liver function monitoring. The proportional changes in ALBI grade following durvalumab treatment at baseline, and after 4 and 8 weeks. (TIF 242 KB) [file 12072_2024_10728_MOESM4_ESM.tif]
