# Supplementary material for: Efficacy and safety of durvalumab rechallenge in advanced hepatocellular carcinoma patients refractory to prior anti-PD-1 therapy
Source: Hepatol Int. 2024 Nov 23;18(6):1804–14. doi: 10.1007/s12072-024-10728-9 (PMC11632046; doi:10.1007/s12072-024-10728-9)
Supplement: Supplementary file 5 — Supplementary file5 (DOCX 31 KB) [file 12072_2024_10728_MOESM5_ESM.docx]

Supplementary table 1. Patient characteristics

| Factors | Case numbers | % |
| --- | --- | --- |
| Age (Mean+/- SD) | 60.5 +/-11.6 |  |
| M/F | 48/17 | 73.8%/26.2% |
| Etiology |  |  |
| HBV | 28 | 43.1% |
| HCV | 2 | 3.1% |
| Non-viral | 35 | 53.8% |
| Laboratory data | Median | IQR |
| Platelet count (10^9/L) | 155.0 | (109.5－220.8) |
| ALT (U/L) | 37.0 | (28.0－54.0) |
| ALB (g/dL) | 3.8 | (3.5－4.0) |
| Total bilirubin (mg/dL) | 0.7 | (0.5－1.2) |
| PT (second) | 11.6 | (11.0－12.7) |
| AFP (ng/mL) | 690.0 | (22.8－7563.0) |
| PIVKA-II (mAL/mL) | 4707.0 | (577.5－34639.3) |
| CPS score class | Case numbers | % |
| A | 46 | 76.7% |
| B | 11 | 18.3% |
| C | 3 | 5.0% |
| ALBI grade |  |  |
| I | 25 | 39.1% |
| II | 36 | 56.3% |
| III | 3 | 4.7% |
| Radiological factors |  |  |
| Cirrhosis | 27 | 41.5% |
| Ascites |  |  |
| No | 40 | 61.5% |
| Mild/moderate | 24 | 36.9% |
| Severe | 1 | 1.5% |
| Intra-hepatic tumor numbers |  |  |
| ≤10 | 42 | 64.6% |
| >10 | 19 | 29.2% |
| Infiltrating type | 4 | 6.2% |
| Intra-hepatic tumor size (cm) |  |  |
| ≤10 | 49 | 75.4% |
| >10 | 12 | 18.5% |
| Infiltrating type | 4 | 6.2% |
| Macrovascular invasion | 28 | 43.1% |
| Extrahepatic metastasis | 45 | 69.2% |
| BCLC stage |  |  |
| B/C/D | 14/48/3 | 21.5%/73.8%/4.6% |
| Previous local treatment |  |  |
| Surgical tumor resection | 26 | 40.0% |
| RFA | 12 | 18.5% |
| TACE | 38 | 58.5% |
| Radiotherapy | 9 | 13.8% |
| Lines of prior systemic therapy |  |  |
| 1 | 15 | 23.1% |
| 2 | 27 | 41.5% |
| ≥ 3 | 23 | 35.4% |
| Experienced targeted therapy |  |  |
| Lenvatinib | 55 | 84.6% |
| Sorafenib | 32 | 49.2% |
| Regorafenib | 12 | 18.5% |
| Cabozantinib | 5 | 7.7% |
| Experienced anti-PD-1 |  |  |
| Nivolumab | 56 | 86.2% |
| Pembrolizumab | 9 | 13.8% |
| Combined drugs with durvalumab |  |  |
| Lenvatinib | 41 | 63.1% |
| Regorafenib | 11 | 16.9% |
| Sorafenib | 4 | 6.2% |
| Cabozantinib | 4 | 6.2% |
| Ramucirumab | 2 | 3.1% |
| Bevacizumab | 1 | 1.5% |
| Chemotherapy | 1 | 1.5% |

IQR = interquartile range; ALT: Alanine Aminotransferase; ALB: Albumin; PT: Prothrombin Time; AFP: Alpha-Fetoprotein; PIVKA-II: Protein Induced by Vitamin K Absence or Antagonist-II; CPS score class: Child-Pugh Score class; ALBI grade: Albumin-Bilirubin grade; BCLC stage: Barcelona Clinic Liver Cancer stage; RFA: Radiofrequency Ablation; TACE: Transarterial Chemoembolization

Supplementary table 2. Therapeutic efficacy of different combination regimens with durvalumab

| Combined regimens | Case numbers | Overall response rate |
| --- | --- | --- |
| Durvalumab plus lenvatinib | 41 | 9.8% |
| Durvalumab plus regorafenib | 11 | 36.4% |
| Durvalumab plus sorafenib | 4 | 25.0% |
| Durvalumab plus cabozantinib | 4 | 0% |
| Durvalumab plus ramucirumab | 2 | 0% |
| Durvalumab plus bevacizumab | 1 | 0% |
| Durvalumab plus chemotherapy | 1 | 0% |
| Durvalumab monotherapy | 1 | 0% |

Supplementary table 3. Incidence of irAE associated with durvalumab

|  | irAE grading | | | | |
| --- | --- | --- | --- | --- | --- |
| Organ | Grade 1 | Grade 2 | Grade 3 | Grade 4 | All |
| Skin toxicity | 5 (7.7%) | 3 (4.6%) | 0 | 1 (1.5%) | 9 (13.8%) |
| Hypothyroidism | 1 (1.5%) | 0 | 0 | 1 (1.5%) | 2 (3.1%) |
| Hepatitis | 3 (4.6%) | 2 (3.1%) | 0 | 0 | 5 (7.7%) |
| Pneumonitis | 0 | 0 | 0 | 1 (1.5%) | 1 (1.5%) |
| Encephalopathy | 0 | 1 (1.5%) | 0 | 0 | 1 (1.5%) |
| Diarrhea | 0 | 0 | 1 (1.5%) | 0 | 1 (1.5%) |
| Fever | 0 | 2 (3.1%) | 0 | 0 | 2 (3.1%) |
| Diabetes mellitus | 0 | 0 | 0 | 1 (1.5%) | 1 (1.5%) |
| Pancreatitis | 0 | 0 | 1 (1.5%) | 1 (1.5%) | 2 (3.1%) |
| Myalgia | 0 | 0 | 1 (1.5%) | 0 | 1 (1.5%) |

irAE, immune-related adverse event

Supplementary table 4. Association of irAEs with prior anti-PD-1 and subsequent durvalumab

|  | irAE of durvalumab | | *p* value |
| --- | --- | --- | --- |
| Grading | 0-2 | ≧3 |  |
| irAE of prior anti-PD-1 |  |  |  |
| 0-2 | 56 (93.3 %) | 4 (6.7 %) | 1.0 |
| ≧3 | 5 (100 %) | 0 (0 %) |  |

irAE, immune-related adverse event

Supplementary table 5. Association of irAEs with prior anti-PD-1 and subsequent durvalumab

|  | irAE of durvalumab | | *p* value |
| --- | --- | --- | --- |
| Grading | 0 | Any |  |
| irAE of prior anti-PD-1 |  |  |  |
| 0 | 31 (70.5 %) | 13 (29.5 %) | 0.63 |
| Any | 16 (76.2 %) | 5 (23.8 %) |  |
